# Microbial Community Composition and Activity in Saline Soils of Coastal Agro–Ecosystems

**DOI:** 10.3390/microorganisms10040835

**Published:** 2022-04-18

**Authors:** Yang Dong, Jianwei Zhang, Ruirui Chen, Linghao Zhong, Xiangui Lin, Youzhi Feng

**Affiliations:** 1 State Key Laboratory of Soil and Sustainable Agriculture, Institute of Soil Science, Chinese Academy of Sciences, Nanjing 210008, China; dongyang@issas.ac.cn (Y.D.); zhangjianwei@issas.ac.cn (J.Z.); xglin@issas.ac.cn (X.L.); yzfeng@issas.ac.cn (Y.F.); 2 University of Chinese Academy of Sciences, Beijing 100049, China; 3 Jiangsu Collaborative Innovation Center for Solid Organic Waste Resource Utilization, Nanjing 210095, China; 4 Mont Alto 1 Campus Drive, Pennsylvania State University, Mont Alto, PA 17237, USA; luz4@psu.edu

**Keywords:** soil salinity, microbial richness, microbial metabolic activity, organic C, microbial community composition

## Abstract

Soil salinity is a serious problem for agriculture in coastal regions. Nevertheless, the effects of soil salinity on microbial community composition and their metabolic activities are far from clear. To improve such understanding, we studied microbial diversity, community composition, and potential metabolic activity of agricultural soils covering non–, mild–, and severe–salinity. The results showed that salinity had no significant effect on bacterial richness; however, it was the major driver of a shift in bacterial community composition and it significantly reduced microbial activity. Abundant and diverse of microbial communities were detected in the severe–salinity soils with an enriched population of salt–tolerant species. Co–occurrence network analysis revealed stronger dependencies between species associated with severe salinity soils. Results of microcalorimetric technology indicated that, after glucose amendment, there was no significant difference in microbial potential activity among soils with the three salinity levels. Although the salt prolonged the lag time of microbial communities, the activated microorganisms had a higher growth rate. In conclusion, salinity shapes soil microbial community composition and reduces microbial activity. An addition of labile organic amendments can greatly alleviate salt restrictions on microbial activity, which provides new insight for enhancing microbial ecological functions in salt–affected soils.

## 1. Introduction

Soil salinity is deemed as a worldwide environmental challenge threatening cultivated land area and crop productivity. It has been estimated that worldwide 20% of total cultivated and 33% of irrigated agricultural lands are afflicted by soil salinity [1]. It is estimated that 50% of all arable land will become impacted by primary and secondary salinization by 2050 [2]. The loss of farmable land and decreased yield is in direct conflict with the needs by the growing world population. Restoration of salinized lands is an indispensable component for feeding the world [3].

Coastal saline soil is a common form of soil salinization. In China, coastal saline land is mainly distributed in the west coast of the Bohai Sea and the provinces along the southeast coast. As most of these places are economically developed areas with a large population and marginal arable land, exploitation and utilization of coastal saline land as potential cropland is urgent in China. As a key ecosystem at the continent–ocean interface, coastal saline soil is rich in continental and marine nutrient inputs [4]. Even so, it cannot undertake normal soil processes because high soil salinity significantly inhibits microbial ecological function (activity), specifically reflected in microbial participation in elemental transformation [5]. Of course, microbial activity in salt–affected soils is not static due to soil salinity is greatly fluctuated with temperature (evaporation) and precipitation [6]. As microbial diversity plays an important role in maintaining microbial ecological multifunctionality [7], the effect of soil salinity on microbial diversity deserves particular attention. A meta–analysis based on 111 global studies suggested that salinity rather than pH, temperature or other physiochemical environmental factors dominate microbial community composition [8]. However, the response of microbial diversity to increasing salinity in agricultural soils (limited salinization) is not yet fully clear. In addition to microbial community structure, salinity stress can also change interactions between species [9]. There is growing evidence that the functioning and stability of bacterial communities are greatly affected by this interaction [10]. By analyzing microbial richness, community composition, and molecular ecology networks, we can better understand the succession of microbial communities due to soil salinity.

Nowadays, more and more agricultural and soil scientists rely on studying soil microbial activity to evaluate the health and sustainability of soil ecosystems [11]. Jeppesen et al. [12] found that microbial activity follows a non–linear response to salinity, where minor salinity changes beyond the threshold will cause drastic fluctuations in microbial activity. Rath and Rousk [13] proposed that the direct negative effects of salinity on microbes might not be the only reason for inhibited microbial activity. The inhibition could also be attributed to the lack of organic matter (OM) input associated with the sparse plant growth in saline soils. Although it is known that adding exogenous organic matter to saline soils can significantly improve microbial activity [14], little is known about the growth response of microbial communities with C input in saline soils. Microbial strategies to assimilate C for maintenance and growth will provide useful information on physiological mechanisms of microbial resistance to environmental stress. Traditional methods for studying soil microbial activity were mainly soil respiration [15] and enzymatic activities [16]. Recently, an isothermal microcalorimetric analysis is of particular interest for studying microbial metabolic processes in soil carbon dynamics [17], with the advantages of high precision, and high data frequency compared to CO_2_ measurements. Being a non–intrusive method, it can reveal the kinetics of microbial growth in response to glucose addition.

To test whether C is another inhibitor of microbial activity in salinized soils, we collected a series of soil samples in the coastal area of the Bohai Sea. The microbial richness (observed by high throughput sequencing) and metabolic activities (characterized by microcalorimetry) were evaluated and analyzed. The objective of this study was (1) to assess the effect of soil salinity on alpha and beta diversity of soil bacterial communities, as well as their co–occurrence patterns, and (2) to test whether the addition of labile C can improve the metabolic activity of microbial communities with salinity. The insights gained from this study will improve our understanding of the effects of salinity on soil microorganisms. If proven true, our hypotheses can provide a guideline for saline soils restoration by the amendment of organic matters.

## 2. Materials and Methods

### 2.1. Sites

Soil samples were collected in the coastal area of Sino–Czech–Slovak Friendship Farm (37°24′, 117°31′) in the west of the Bohai Sea and southeast of Hebei Province, China. The area has a temperate monsoon climate influenced by Eurasia and the Pacific Ocean. The mean annual temperature is 12.5 °C and the mean annual rainfall is 581 mm (obtained from http://www.weather.com.cn (accessed on 13 February 2022)). The farm is mostly monocropped with summer maize (*Zea mays* L.) and winter fallow. The average production of maize in this region is 6–7.5 t ha^−1^, which is remarkably lower than the 7–10 t ha^−1^ of the North China Plain average production [18].

Samples were collected on 20 August 2017, about one month before the maize harvest. There was no precipitation for half a month before sample collection. Ten survey sites (30 m × 30 m) were selected with six sampling points along a diagonal in each site. At each sampling point, 6 cores were taken at a depth of 0–10 cm using a 30 mm diameter gouge auger. The 6 cores of soil were mixed, coarse roots and stones were removed and then taken to the laboratory on ice. Each soil sample after being sieved (<2 mm) was split into two parts. One subsample was immediately stored at −40 °C for DNA extraction, and the other subsample was air–dried, sieved and used for the analysis of chemical properties.

### 2.2. Analysis of Soil Chemical Properties

Soil chemical properties were analyzed according to the protocols of Lu [19]. Soil EC was determined from soil–water suspensions (1: 5 *v*/*v*). Soil pH was determined from soil–water suspensions (1:2.5 *v*/*v*). Soil organic C (SOC) was determined by potassium dichromate oxidization and back titration of excess potassium dichromate using an ammonium ferrous sulphate solution. Available N (AN) in the soil was alkaline–hydrolyzed and produced NH_3_ was diffused and determined by acid–base neutralization titration. Available P (AP) in the soil was extracted by sodium bicarbonate and determined using the molybdenum blue method. The content of potassium (K) and sodium (Na) in the soil was extracted by ammonium acetate and determined by flame photometry. Soil dehydrogenase activity was determined by the reduction of triphenyltetrazolium chloride (TTC) to triphenylformazan (TPF) as described by Chu et al. [20].

Soil EC of ten plots ranged from 244 to 4374 μS/cm (Table 1). Salinity contents were calculated using the conversion method between salinity and EC (Equation (1)) obtained from coastal saline areas [21].

(1) $$\mathrm{salinity}=\left(\frac{\mathrm{EC}}{1000}-0.0182\right)/0.39$$

where the unit of salinity was g/kg and the unit of EC was μS/cm.

Based on the salinity threshold [21], ten plots were classified into three groups, Non–salinity (Plot 3, 5 and 8 with salinity less than 1 g/kg), Mild–salinity (Plot 1, 4, 6 and 10 with salinity between 1 and 4 g/kg) and Severe–salinity (Plot 2, 7 and 9 with salinity greater than 4 g/kg).

### 2.3. DNA Extraction and High–Throughput Sequencing of 16S rRNA Genes

Genomic DNA was extracted from 0.5 g soil by using a FastDNA SPIN Kit for soil (MP Biomedicals, Santa Ana, CA, USA). The extracted DNA was dissolved in 50 μL TE buffer, quantified by spectrophotometer and quality evaluated by gel electrophoresis. After that, extracted DNA was evaluated by NanoDrop ND–2000 (Thermo Fisher, Waltham, MA, USA) and stored at −20 °C until further usage.

PCR amplification was conducted for bacteria with primer set 519F/907R [22]. The oligonucleotides of 5 bp bar–coded were fused to the forward primer. PCR was carried out in 50 μL reaction mixture, containing deoxynucleotide triphosphate at a concentration of 1.25 μM, 2 μL (15 μM) forward and reverse primers, 2 μM of Taq DNA polymerase (TaKaRa, Japan), and each reaction mixture received 1 μL (50 ng) of genomic community DNA as a template. PCRs were performed according to the following program: 94 °C for 5 min, 30 cycles (94 °C for 30 s, 55 °C for 30 s, 72 °C for 45 s), and a final extension at 72 °C for 10 min. Reaction products for each soil sample were pooled and purified using the QIAquick PCR Purification Kit (Qiagen, Valencia, CA, USA), and quantified using NanoDrop ND–2000 (Thermo Scientific, Waltham, MA, USA).

High–throughput sequencing was performed with Illumina MiSeq sequencing platform (Illumina Inc., San Diego, CA, USA). The bar–coded PCR products from all samples were normalized in equimolar amounts before sequencing. After sequencing was completed, 16S rRNA gene data were processed using the Quantitative Insights Into Microbial Ecology (QIIME) pipeline for data sets (http://qiime.source.org (accessed on 15 June 2019)) [23]. Sequences with a quality score below 25 and a length fewer than 200 bp were trimmed and then assigned to soil samples based on unique barcodes. A total of 796,222 high quality sequences were obtained (max = 24,663, min = 4504, SD = 6549). Sequences were binned into operational taxonomic units (OTUs) using a 97% identity threshold and the most abundant sequence from each OTU was selected as a representative sequence. Taxonomy was then assigned to OTUs with reference to a subset of the SILVA 119 database (http://www.arb-silva.de/download/archive/qiime/ (accessed on 23 June 2019)). A phylogenetic tree was constructed with FastTree using a multiple–sequence alignment made with PyNAST [23]. All samples were then rarefied to 4500 sequences per sample to evaluate beta diversity of phylotypes, which allowed us to compare general diversity patterns among treatments, even though it is highly unlikely that we surveyed the full extent of diversity in each community.

### 2.4. Molecular Ecological Network Analysis

Changes in phylogenetic molecular ecological networks (pMENs) were evaluated using the random matrix theory (RMT) based network approach [24]. The pMEN construction and analyses were performed following the online pipeline (http://129.15.40.240/mena/ (accessed on 20 August 2021)) of Deng et al. [25]. The network graphs were then visualized using Gephi software [26]. Network parameters, such as density, average centralization of degree, transitivity, average degree and average path distance, etc., were used to evaluate the co–occurrence network topological structure.

### 2.5. Microcalorimetric Analysis

Soil microbial activity with and without the addition of glucose was evaluated by microcalorimetric analysis [27,28]. Before the measurement, all soil samples were pre–incubated with 60% WHC at 28 °C in the dark for 24 h. For the measurement without glucose addition, i.e., soil basal heat release, one gram soil was added to a 4 mL glass ampoule and the ampoules were submitted to the microcalorimetric analysis. For the measurement with glucose addition, one–gram pre–incubated soil was added to a 4 mL glass ampoule. 80 μL solution containing 2 mg glucose, 0.19 mg (NH_4_)_2_SO_4_, 0.39 mg K_2_HPO_4_ and 0.76 mg MgSO_4_•7H_2_O was added to each soil sample as substrates. The ampoules were then immediately placed into an isothermal calorimetric monitor TAM III (TA Instruments, New Castle, DE, USA), and the thermodynamics of heat release from the soil samples were continuously monitored and recorded as a power–time curve from the exothermic metabolic reaction.

The thermodynamic parameters derived from the power–time curves are used to evaluate microbial metabolic activity. For the measurement without glucose addition, soil basal heat release (P_bas_) was calculated by the average of heat flow rates during the incubation and expressed as μW g^−1^. A higher basal heat release represents a higher basal metabolic activity of soil microbial communities.

For the measurement with glucose addition, the power–time curves present typical microbial growth curves, with the lag phase, exponential phase, stationary phase and decline phase. Characteristic parameters can be obtained from the curve: peak power (P_max_, μW g^−1^), the maximum heat flow rate; peak power time (T_max_, min), the time when heat flow hits the maximum of the curve. Microbial characteristics of growth response were estimated by fitting the kinetics of substrate–induced heat release with Equation (1). The adapted equation was originally used to estimate the kinetics of microbial growth based on measured substrate–induced CO_2_ evolution rate [29,30].

(2) $$P=A+B*\exp\left(k*t\right)$$

where A is the initial heat flow rate uncoupled from cell growth (μW g^−1^); B is the initial heat flow rate of the growing fraction coupled with cell growth (μW g^−1^); k (h^−1^) is the specific growth rate of the soil microbial community, and t is the time. The parameters of Equation (2) were optimized by minimizing the least–square sum using ‘microcalorimetry’, which is a self–built R package (https://github.com/zw-jing/microcalorimetry (accessed on 20 August 2021)). Fitting was restricted to the initial phase of the curve, corresponding to unlimited exponential growth.

### 2.6. Calculations and Statistical Analysis

The phylogenetic diversity (PD score) and Richness were calculated by ‘picante’ and ‘vegan’ packages of R 3.6.1, respectively, to evaluate the bacterial phylogenetic diversity and taxonomic richness. Based on ‘vegan’ package, the dissimilarities of the bacterial community composition were calculated based on the Bray–Curtis distance, which was visualized by nonmetric multidimensional scaling analyses (NMDS) and tested for significance by PERMANOVA. Redundancy analysis (RDA) was carried out to determine the effect of soil properties on the bacterial community using ‘vegan’ package. Mantel test was conducted by ‘vegan’ package to compare the relative impacts of soil chemical properties on microbial community. Random forest (RF) mean predictor was conducted by ‘randomForest’ package to unravel species discriminating microbial community composition. ANOVA was performed to determine the effects of salinity on soil chemical and microbial properties. Mean separation was carried out based on Tukey’s HSD test. The statistical analysis was performed using SPSS version 19.0 for Windows (SPSS Inc., Chicago, IL, USA). The phylogenetic tree was visualized by the webtool iTOL (Interactive Tree of Life (https://itol.embl.de/ [accessed on 8 April 2022])). Other plots were completed using ‘circlize’ and ‘ggplot2′ packages of R 3.6.1.

## 3. Results

### 3.1. Soil Chemical Properties and Dehydrogenase Activities

**Figure 1:**
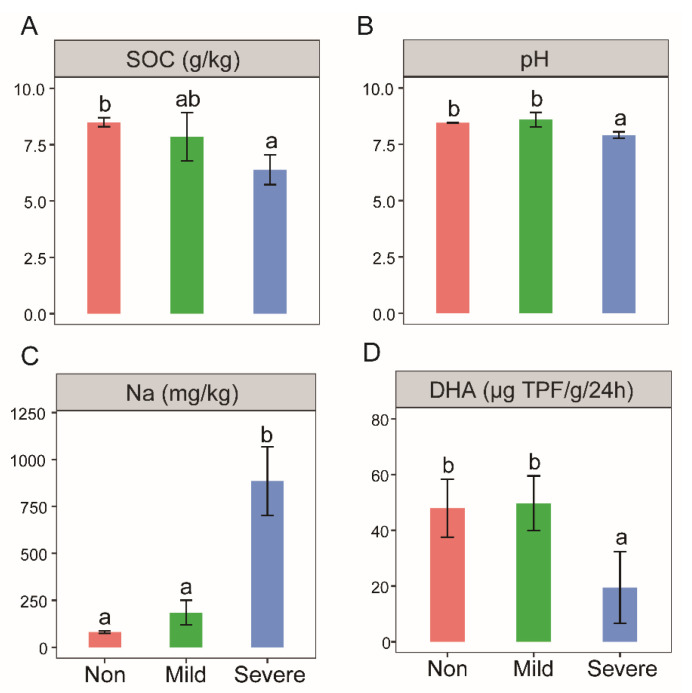
SOC contents (**A**), pH (**B**), Na contents (**C**) and dehydrogenase activities (**D**) of the soils with three salinity levels: Non–salinity, Mild–salinity and Severe–salinity. Different letters above bars denote significant differences (*p* < 0.05) among salinity levels.

The SOC and pH under Severe–salinity soils were significantly lower than those under Mild– and Non–salinity soils (Figure 1A,B). Na content with Severe–salinity was significantly higher, roughly 10 times higher than with Non–salinity, and about five times higher than Mild–salinity (Figure 1C). These two indices are not significantly different between Mild– and Non–salinity. Besides, there was a significant negative linear correlation between soil EC and soil pH (R^2^ = 0.64, *p* < 0.01), and a significant positive linear correlation between soil EC and Na content (R^2^ = 0.74, *p* < 0.01).

Soil dehydrogenase activity was significantly inhibited by Severe–salinity, which was less than half of activities with Mild– and Non–salinity (Figure 1D). No significant difference was observed between soils with Mild– and Non–salinity. In addition, there was a significant positive linear correlation between soil dehydrogenase activity and SOC content (R^2^ = 0.53, *p* < 0.05).

### 3.2. Bacterial Community Richness and Composition

In total, 3139 OTUs were identified and assigned to 24 phyla, 60 classes and 116 orders. Of these OTUs, 99.49% (3123 OTUs) were bacterial, and the rest (16 OTUs) was affiliated with the Archaea. Five bacterial phyla (Proteobacteria, Actinobacteria, Acidobacteria, Chloroflexi and Bacteroidetes) were dominant phyla accounting for 82.45% of the total sequences (Figure S1). Among them, the relative abundance of Proteobacteria was the highest, which was 28.07%. Followed were Actinobacteria (20. 55%) and Acidobacteria (19.36%). Acidobacteria in Severe–salinity soils was significantly lower than that in Non–salinity soils, while Bacteroidete was the opposite (*p* < 0.05, Figure S1).

**Figure 2:**
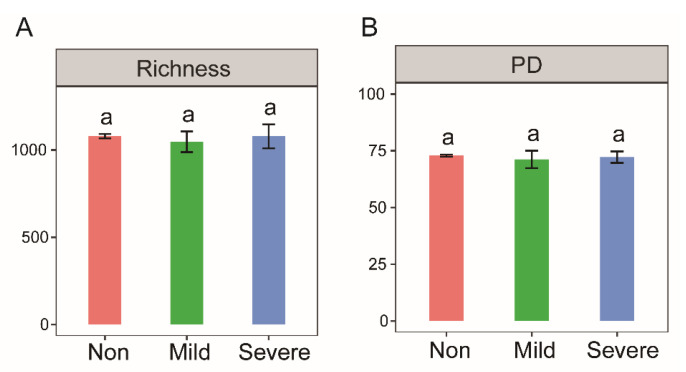
Bacterial diversity indices of the soils with three salinity levels: Non–salinity, Mild–salinity and Severe–salinity. The diversity index Richness denotes the bacterial variations at the taxonomic level (**A**), and PD (phylogenetic diversity) denotes variations at the phylogenetic level (**B**), in other words, evolutionary relationship. The same letters above bars denote no significant difference (*p* > 0.05) among salinity levels.

By constructing a phylogenetic tree, we found that phylogenetic diversity (PD) was not visually different among the three salinities (Figure S2). The PD score (Figure 2B) and Richness index (Figure 2A) further suggested that salinity has no significant effect on bacterial diversity. In addition, there was no significant correlation between soil dehydrogenase activity and two bacterial diversity indices (*p* > 0.05).

**Figure 3:**
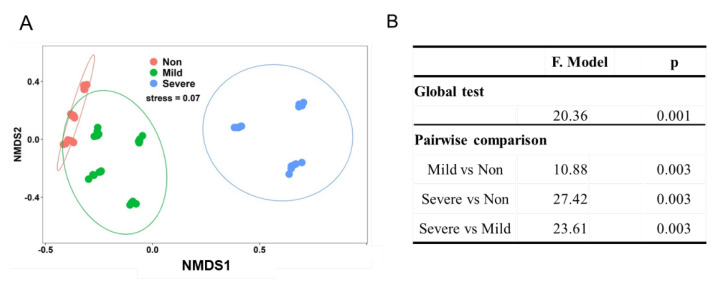
Nonmetric multidimensional scaling (NMDS) plot of bacterial community in the soils with three salinity levels: Non−salinity, Mild−salinity and Severe−salinity. Plot (**A**) depicts the Bray–Curtis distance and PERMANOVA with a global test and pairwise comparison (**B**) shows Bray–Curtis distance−based dissimilarity of the bacterial community in the soils with three salinity levels.

**Figure 4:**
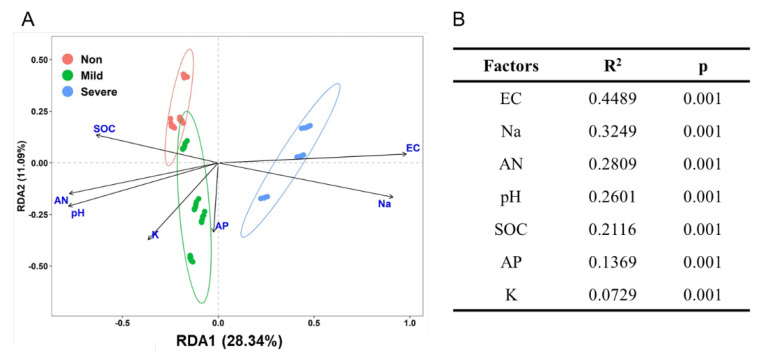
Redundancy analysis (RDA) plot of the soils with three salinity levels: Non−salinity, Mild−salinity and Severe−salinity. Plot (**A**) depicts the correlation between bacterial community and soil chemical properties and Mantel test (**B**) compares the relative impacts of soil chemical properties on the bacterial community. EC: electrical conductivity; SOC, soil organic C; AP: available P; AN: available N.

Nonmetric multidimensional scaling analyses (NMDS) revealed that soil samples formed distinct clusters in the ordination space based on Bray–Curtis distance (Figure 3A), with significant differences at taxonomic levels (PERMANOVA) (Figure 3B). It is obvious that Non– and Mild–salinity soils were clustered close and they were distant from the cluster of Severe–salinity soils. We can conclude that soil salinity significantly influenced the composition of the bacterial community, which was also confirmed in ordination analysis (Figure 4A) and Mantel test (Figure 4B). From redundancy analysis, we found approximately 80.32% of the total variation in the soil bacterial community composition with the first two components explained 39.43% (Figure 4A). The effects of EC and Na on bacterial community were opposite to that of SOC, AN and pH. Mantel test showed that EC was the major driver of a shift in bacterial community composition, explaining 44.89% of the variation (*p* = 0.001, Figure 4B).

**Figure 5:**
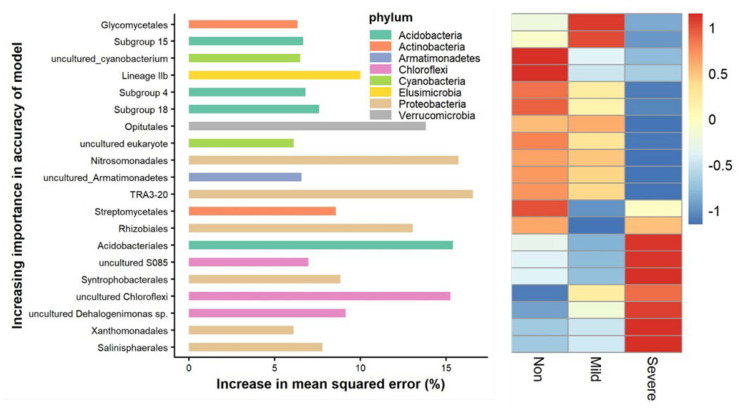
Random forest (RF) means predictor importance (percentage of increase in mean square error) of microbial species at order level and the corresponding relative abundances (z–score transformed) of the soils with three salinity levels: Non–salinity, Mild–salinity and Severe–salinity. The accuracy importance of the model was computed for each tree and averaged over the forest (1000 trees). Percentage increases in the mean squared error (MSE) of variables were used to estimate the importance of these predictors, and higher values indicate more important predictors. Heatmap showed the variations in the relative abundances of these top 20 predictive biomarker taxa among three salinity soils.

To unravel the biomarker species discriminating the bacterial community compositions along the salinity gradient, we performed a regression analysis of the relative abundances of bacterial species at the order level with three salinity levels. The top 20 most important orders were selected as the respective biomarker taxa (Figure 5). Heatmap of relative abundances further indicated that the most important biomarker species for salinity–sensitive species were largely affiliated with TRA3–20, Nitrosomonadales in the phylum of Proteobacteria, Opitutales in the phylum of Verrucomicrobia, subgroups in the phylum of Acidobacteria and uncultured Cyanobacteria. In comparison, the most important biomarker species for salinity–tolerant species were largely affiliated with Syntrophobacterales, Xanthomonadales and Salinisphaerales in the phylum of Proteobacteria; Acidobacteriales in the phylum of Acidobacteria and uncultured Chloroflexi.

### 3.3. The Influence of Salinity on Bacterial Co–Occurrence Network

**Figure 6:**
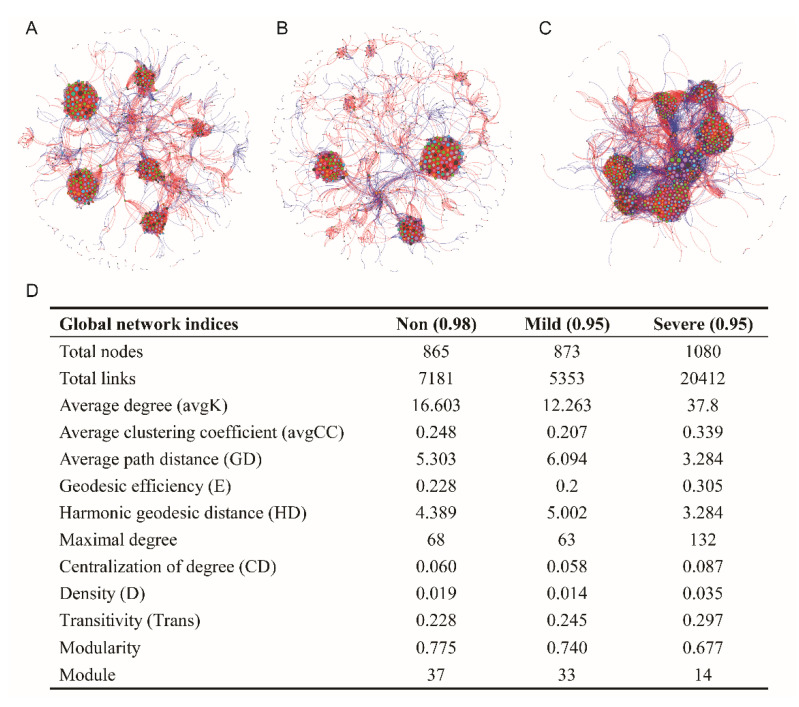
Bacterial network associations (**A**: Non–salinity; **B**: Mild–salinity, **C**: Severe–salinity) and topological properties of bacterial molecular ecological networks (**D**) of soils with three salinity levels. Network plots were drawn based on random matrix theory (RMT) analysis from OTU profiles. Red and blue lines respectively represent negative and position correlations between nodes. The size of each node is proportional to the number of connections with other nodes in the network.

Phylogenetic molecular ecological networks (pMENs) were generated to delineate the influence of salinity on the bacterial co–occurrence network (Figure 6). Permutation tests indicated that all the observed network indices were significantly different from the corresponding random counterpart (Table S1), indicating the observed network indices can characterize the network properties under each saline level. In addition, topological properties of networks were pairwise compared among salinity levels (Table S2).

The network of the Mild–salinity soil had a quite similar pattern to Non–salinity soil (Figure 6A,B,D). In contrast, the network of the Severe–salinity soil had 25% more nodes and 184% more links than those of the Non–salinity and Mild–salinity soils. Average path distance and geodesic distance significantly decreased with Severe–salinity, while average degree and transitivity significantly increased with higher Severe–salinity (*p* < 0.01, Table S2). The value of density and degree of centralization was also higher with severe–salinity, although without available statistical analysis (Table S2). All of these findings unanimously suggested that severe salinity complicated and centralized the bacterial co–occurrence network.

### 3.4. Soil Microbial Activities Revealed by Microcalorimetry

**Figure 7:**
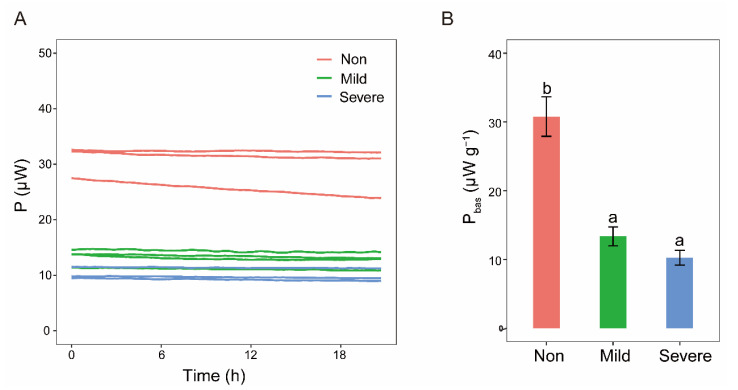
Basal heat release curves (**A**) and average basal heat release P_bas_ with the unit of μW g^−1^ (**B**) of the soils with three salinity levels: Non–salinity, Mild–salinity and Severe–salinity. Different letters above bars denote significant differences (*p* < 0.05) among salinity levels.

Recorded power–time curves of basal heat release were almost parallel to the *x*–axis (Figure 7A), indicating that microbial activities were relatively stable when microorganisms were in the state of maintenance without substrates amendment. The basal heat release curves visually reflected that microbial metabolic activity in the soils with three salinity levels had an order of Non–salinity > Mild–salinity > Severe–salinity (Figure 7A). Analysis of one–way ANOVA further indicated that basal heat release of Non–salinity soil was significantly higher than that of Mild–salinity soil and Severe–salinity soil (Figure 7B, *p* < 0.05).

**Figure 8:**
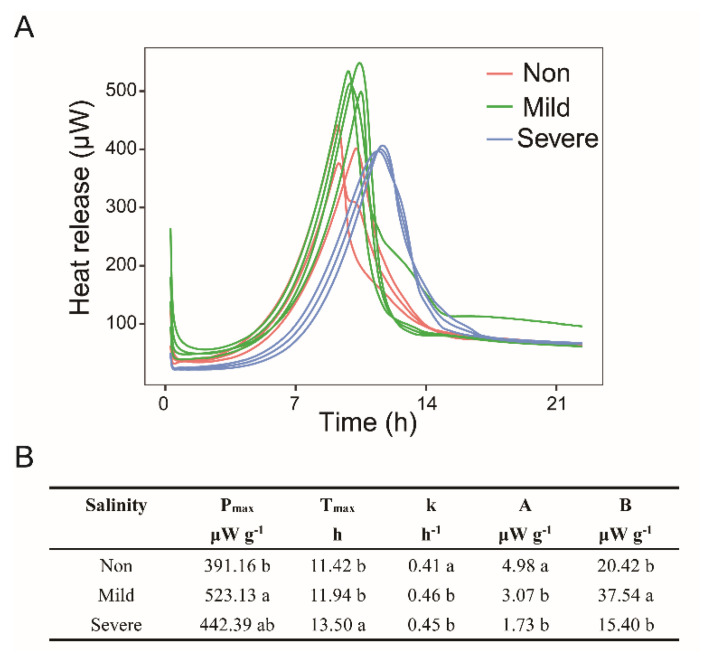
Substrate induced heat release curves (**A**) and microbial characteristics of growth response with three salinity levels (**B**): Non–salinity, Mild–salinity and Severe–salinity. Different letters above bars denote significant differences (*p* < 0.05) among salinity levels. P_max_, the maximum heat flow rate; T_max_, the peak power time; k, the specific growth rate of the soil microbial community; A, the initial heat flow rate uncoupled from cell growth; B, the initial heat flow rate of the growing fraction coupled with cell growth.

The thermodynamics of substrate–induced heat release curves visually reflected the regular change in microbial growth with the increase in soil salinity (Figure 8A). Analysis of one–way ANOVA showed that soils with severe salinity showed a significantly increased in T_max_ than in soils with Mild– and Non–salinity (*p* < 0.05, Figure 8B). It suggests that soil microbes have a longer lag time and delayed the pulse of heat release under severe salinity. However, P_max_ had an order of Mild–salinity > Severe–salinity > Non–salinity, with a significant difference between Mild–salinity and Non–salinity (*p* < 0.05, Figure 8B). The specific growth rate of the soil microbial community was observed to be the lowest in Non–salinity soils (*p* < 0.05, Figure 8B), and significantly lower than in soils with Mild–salinity and Severe–salinity (*p* < 0.05, Figure 8B).

## 4. Discussion

### 4.1. Microbial Community Composition in Response to Soil Salinity

Conventionally, diversity has been considered a desirable characteristic, reflecting an ecosystem’s ability to provide complete functions [31,32]. Our statistical analysis demonstrated a similar bacterial richness among all three salinity levels (Figure 2), indicating the current salinity does not affect the richness of the microbial community. It turned out our finding is not completely isolated. A similar result was also reported for archaeal diversity along a soil salinity gradient in British Columbia, Canada [33]. Canfora et al. [34] found higher species richness and Shannon–Wiener index at high levels of salinity from a coastal system in central Italy. In addition, surprisingly abundant and diverse bacterial communities have been discovered in extreme saline environments, harboring a wide variety of taxa [35,36,37]. Such abundant and diverse communities could be attributed to the long–term determination of historical effects, as these study sites are naturally saline habitats including salt plains [36], rock–salt deposits [37] and hypersaline lakes [35]. In theory, the survival strategies of microorganisms and the assembly mechanisms of microbial communities are largely determined by their historical biotic and abiotic conditions [38]. From recent studies of coastal saline soils, we believe most species could have originated from marine environments, and can therefore maintain a strong adaptation to soil salinity; thus, severe salt stress does not decrease soil bacterial diversity. On the contrary, a short–term salinity increase can severely reduce the richness of the microbial community, as shown by an anaerobic membrane bioreactor with NaCl addition [39].

The relationship between the alpha diversity and salt content is also influenced by the value of salt content. The relationship was reported to be a one–humped curve based on data from the hypersaline Ebinur Lake shoreline, in which phylotype richness and phylogenetic diversity were generally higher in soils with salt content between 3.5 and 5.5%, while lower with the low salt content of 1.5–2.5% and high content of 6.5–7.5% [40]. Both the increase and decrease in alpha diversity could be possible with the increase in salt content. The inflection of salt content could be variable as a result of many factors in a certain ecosystem.

Albeit having similar alpha diversity to bacteria, the statistical comparisons (NMDS and RDA) revealed a significant shift in bacterial community composition along the salinity gradient (Figure 3 and Figure 4). Our results agreed with many previous studies that soil salinity, represented as EC and Na, significantly affected the composition of bacteria [35,41,42] and archaea [33]. Such a shift is attributed to the salt selection exerted on organisms, rather than nutrient status [43]. More salt–tolerant organisms are favored in such environments, leading to a shift towards a more tolerant community [13]. Firmicutes are usually considered the special indicators in high salinization soils [44], while we found that Bacteroidetes are significantly enriched in Severe–salinity soils (Figure S1). Although salt significantly reduced the relative abundance of Acidobacteria, it was still the main inhabitant in Severe–salinity soils. The result showed that Proteobacteria was the most dominant in Mild– and Severe–salinity soils (Figure S1) indicating that Proteobacteria is salt tolerant to some extent. Alcanivorax borkumensis, a salt–tolerant species of Proteobacteria with oil–degrading capabilities [45], played an important role in cleaning up oil spills in the Mediterranean Sea off Alaska and Spain. At the species level, Syntrophobacterales, Xanthomonadales and Salinisphaerales in the phylum of Proteobacteria; Acidobacteriales in the phylum of Acidobacteria; and uncultured Chloroflexi were enriched with Severe–salinity (Figure 5). Many of them have been reported to be salt–tolerant phylotypes. For example, all members of Salinisphaerales are isolated from marine/oceanic and high–salinity environments and they are halophilic or halotolerant [46]; and syntrophobacterales, anaerobic sulfate–reducing bacteria, are rich in salt–marsh sediments that have high levels of salt and sulfur [47]. While these salt–tolerant organisms have become more populated, some salinity–sensitive species (e.g., Nitrosomonadales, Opitutales, etc.) have become rarer, due to the stronger filtering effect on the bacterial communities by the higher soil salinity [48]. Nitrosomonadales have been previously reported to have a negative response to soil salt content in the marsh ecosystem [49].

### 4.2. Microbial Network in Responses to Soil Salinity

The co–occurrence ecological network analysis is now widely used to identify direct or indirect potential biotic interactions between microbial phylotypes, habitat affinities, or shared physiologies beyond the composition and diversity metrics [50,51]. A recent study reported that environmental stress could destabilize microbial networks [9]. In this case, the developed ecological molecular networks manifested that total nodes, total links, network density and the degree of centralization were higher with Severe–salinity, compared to Non–salinity and Mild–salinity (Figure 6). It revealed the complexity and centralization of the microbiome with Severe–salinity. It was proposed that microbes can maintain ecosystem functions by enhancing interactions between species [52] and by avoiding cascading collapses of the interspecies network in the presence of salinity [53,54]. The positive link percentage was reported to be over 98% in saline soils, suggesting bacteria can share some common strategies and intensify their cooperation to minimize the salt stress [55]. A similar finding was also reported in an aquatic ecosystem where mutualistic interactions of microbial communities showed an increase along the salinization [43]. Hesse et al. [56] similarly found that toxic copper stress obviously increased positive interactions between species. The ecological molecular networks also showed that module numbers were obviously lower with Severe–salinity than with Non– or Mild–salinity (Figure 6). Assuming the number of network modules is a gauge of potential ecological functions, the higher number of modules found under severe–salinity implies fewer microbial ecological functions. When microorganisms are exposed to high stress, or when accessible resources are limited, they have to coexist, and each gives up some functions for survival [57]. In salinity soils, although the low diversification of functional traits of a microbial community is not the most optimal for its stability and sustainability [58,59], increases in cell–cell dependency among microbial members can tighten their connections and counteract this negative effect.

Needless to say, a great amount of energy is needed to support so many linkages and to maintain such a complex network. In soils with severe salinity, the increased respiration activity, also due to high cell–cell dependency, consumes a significant amount of C, resulting in a lower percentage of C retained as biomass in soil. In terms of a single cell, the most rapid response to salinity, in both Gram–positive and Gram–negative bacteria, is a stimulated potassium uptake, to achieve osmotic equilibrium [60]. Another common adaptation to salinity is to take up suitable compounds (also called osmolytes), which do not interfere with cell metabolism and accumulate them in the cell to counterbalance the osmotic difference [61]. Polyols, sugars, amino acids, and their derivatives, betaines, carnitines and prolines are the major types of osmolytes in bacterial osmoadaptation [62,63]. Both osmotic adaptation strategies are energetically expensive [64] and cause significant C loss. An investigation of hypersaline soils from Bangladesh reported that microbial adaptation to salinity was accompanied by a high metabolic quotient, resulting in lower substrate use efficiency [65]. Consequently, salinity greatly hampers soil C sequestration [66], as also shown by the reduced SOC content in soils with Severe–salinity in our study (Figure 1A).

### 4.3. Microbial Activity in Responses to Carbon

In this study, dehydrogenase activity and microbial basal heat release were observed significantly decreased with Severe–salinity (Figure 1D and Figure 7), which is coincident with previous studies that microbial activity was hampered in salinization soils [67,68]; hence, their low biomass [69,70]. Is salt the only inhibiting factor of microbial activity in saline soil? Among all 51 surveyed sites, no correlation was found between microbial biomass and soil salinity, and soil organic C content was the primary predictor of microbial biomass and rates of C mineralization [71]. As revealed by the thermodynamic results, glucose amendment greatly alleviated the inhibition of salt on microbial activity (Figure 8). The same conclusion was also reported by a previous study that organic matter addition alleviated the negative impact of salinity on microbial respiration and growth [72]. They supported Rath and Rousk’s opinion [13] that the low activity is not only the result of direct negative effects of salinity on microbial communities, but also caused by a C deficiency due to a diminished or even disappeared organic matter input from plants. In addition to low organic carbon input, the large amount of carbon consumed by microorganisms for survival (a detailed discussion was in Section 3.2) also exacerbates the C deficiency. However, the C amendment may not always restore microbial activity in salinization soil. Pathak et al. [73] proposed that adding straw significantly improves microbial activity in salinization soils, but it was still significantly negatively correlated with salinity. The response of microbial activity to salinity followed a nonlinear relationship due to the interaction of SOM and salinity. Below and above the obtained salinity threshold, the main controlling factor of microbial activity shifted from SOM to salinity [74].

Additionally, substrate–induced heat release curves showed that the peak power time T_max_ significantly increased with Severe–salinity (Figure 8B), indicating that it took a long time to recover microbial activity with glucose amendment. Similarly, a previous study reported that high salt concentrations substantially delay the pulse of respiration induced by re–wetting dry soil [75]. There are two possible reasons for the delayed T_max_: the lag period is extended or the subsequent exponential growth rate is decreased. However, it is clearly shown that the specific growth rate of the microbial community (k) was significantly increased in two salinized soils (Figure 8B). Thus, one possibility is left that salinity caused a longer lag time delaying the growth of the microbial community.

Two types of microbial growth response patterns were present after rewetting dry soil [76]. Type I with immediate response showed a linear increase in growth, while Type II with a tardive response had a clear lag period followed by an exponential increase in growth. Type II usually exhibited a higher growth rate than Type I. The soil with harsher stress usually had a lower active microbial biomass and showed a Type II response to rewetting [76,77]. The growth response after glucose amendment in saline soils shows a similar pattern. The microbial community in the Non–salinity soil responded faster to the amended C but had a lower growth rate; on the contrary, microbial community in the Severe–salinity soil responded slower to C, but exhibited a higher growth rate. It is speculated that the higher microbial growth rate of Type II is due to the different amounts of resources allocated to individual cells. Specifically, with the same amount of C addition, fewer C will be allocated to each cell in the Non–salinity soils with high microbial biomass (Figure 7B) than in the two salinized soils with low microbial biomass (Figure 7B).

## 5. Conclusions

High salinity greatly changed soil microbial community composition and strengthened dependencies among species. Microcalorimetric analysis shows that soils with salinity, when being added with glucose, can recover their microbial activity, but with a prolonged lag time and increased growth rate of the microbial community. The evidence from this study suggests that the inhibited microbial activity in saline soil is not only attributed to the lack of microbes, but also to the lack of C input. This finding sheds new insight into improving microbial ecological functions in coastal saline soils.

## Figures and Tables

**Table 1:** Electrical conductivity (EC), calculated salinity and soil classification of 10 plots from Sino–Czech–Slovak Friendship Farm.

Plot No.	EC	Salinity	Classification
	μS/cm	g/kg	
1	491	1.24	Mild
2	2807	7.11	Severe
3	334	0.84	Non
4	429	1.09	Mild
5	290	0.73	Non
6	684	1.73	Mild
7	4374	11.07	Severe
8	244	0.62	Non
9	2483	6.29	Severe
10	753	1.91	Mild

## Data Availability

Sequence data have been deposited in the National Center for Biotechnology Information (NCBI) Sequence Read Archive (SRA) with the accession number PRJNA554571.

## Supplementary Materials

The following supporting information can be downloaded at: https://www.mdpi.com/article/10.3390/microorganisms10040835/s1. Table S1. Topological properties of the observed molecular ecological networks (MENs) and their associated random MENs. Table S2. Pair–wise comparison of network parameters among salinity levels. Figure S1. Phylogenetic distributions of dominant bacterial phyla in soils with three salinity levels, Non–salinity, Mild–salinity and Severe–salinity. Figure S2. Phylogenetic distribution of the 3123 bacteria and their relative abundance among three salinity soils.
