# Supplementary material for: Microbial Community Composition and Activity in Saline Soils of Coastal Agro–Ecosystems
Source: Microorganisms. 2022 Apr 18;10(4):835. doi: 10.3390/microorganisms10040835 (PMC9027772; doi:10.3390/microorganisms10040835)
Supplement: Supplementary file 1 [file microorganisms-10-00835-s001.zip › microorganisms-1679357-supplementary.pdf]

Table S1. Topological properties of the observed molecular ecological networks (MENs) and their associated random MENs

	**Non-salinity**		**Mild-salinity**		**Severe-salinity**	
**Network Indexes**	**Observed Network**	**Random Networks**	**Observed Network**	**Random Networks**	**Observed Network**	**Random Networks**
**Average clustering coefficient (avgCC)**	0.248	0.047 +/- 0.002	0.207	0.055 +/- 0.003	0.339	0.064 +/- 0.002
**Average path distance (GD)**	5.303	2.882 +/- 0.010	6.094	3.091 +/- 0.014	3.852	2.421 +/- 0.005
**Geodesic efficiency (E)**	0.228	0.377 +/- 0.001	0.2	0.350 +/- 0.001	0.305	0.445 +/- 0.001
**Harmonic geodesic distance (HD)**	4.389	2.653 +/- 0.007	5.002	2.856 +/- 0.009	3.284	2.247 +/- 0.003
**Centralization of degree (CD)**	0.06	0.060 +/- 0.000	0.058	0.058 +/- 0.000	0.087	0.087 +/- 0.000
**Centralization of betweenness (CB)**	0.047	0.015 +/- 0.002	0.118	0.020 +/- 0.002	0.052	0.010 +/- 0.001
**Centralization of stress centrality (CS)**	517.933	0.163 +/- 0.012	648.053	0.198 +/- 0.019	115.604	0.163 +/- 0.011
**Centralization of eigenvector centrality (CE)**	0.177	0.094 +/- 0.004	0.161	0.103 +/- 0.007	0.109	0.061 +/- 0.002
**Density (D)**	0.019	0.019 +/- 0.000	0.014	0.014 +/- 0.000	0.035	0.035 +/- 0.000
**Reciprocity**	1	1.000 +/- 0.000	1	1.000 +/- 0.000	1	1.000 +/- 0.000
**Transitivity (Trans)**	0.228	0.059 +/- 0.001	0.245	0.077 +/- 0.001	0.297	0.069 +/- 0.001
**Connectedness (Con)**	0.862	0.982 +/- 0.008	0.908	0.988 +/- 0.007	0.965	0.995 +/- 0.004
**Efficiency**	0.979	0.982 +/- 0.000	0.986	0.987 +/- 0.000	0.965	0.966 +/- 0.000
**Hierarchy**	0	0.000 +/- 0.000	0	0.000 +/- 0.000	0	0.000 +/- 0.000
**Lubness**	1	1.000 +/- 0.000	1	1.000 +/- 0.000	1	1.000 +/- 0.000
**Modularity(fast_greedy)**	0.775	0.189 +/- 0.003	0.74	0.221 +/- 0.003	0.677	0.125 +/- 0.003

Table S2. Pair-wise comparison of network parameters among salinity levels

	Mild vs Non	Severe vs Non	Severe vs Mild
**Average clustering coefficient (avgCC)**	0.0001	0.0001	0.0001
**Average path distance (GD)**	0.0001	0.0001	0.0001
**Geodesic efficiency (E)**	0.0001	0.0001	0.0001
**Harmonic geodesic distance (HD)**	0.0001	0.0001	0.0001
**Centralization of degree (CD)**	\	\	\
**Centralization of betweenness (CB)**	0.0001	0.0001	0.0001
**Centralization of stress centrality (CS)**	0.0001	0.0001	0.0001
**Centralization of eigenvector centrality (CE)**	0.0001	0.0001	0.0001
**Density (D)**	\	\	\
**Reciprocity**	\	\	\
**Transitivity (Trans)**	0.0001	0.0001	0.0001
**Connectedness (Con)**	0.0001	0.0001	0.0001
**Efficiency**	\	\	\
**Hierarchy**	\	\	\
**Lubness**	\	\	\
**Modularity(fast_greedy)**	0.0001	0.0001	0.0001

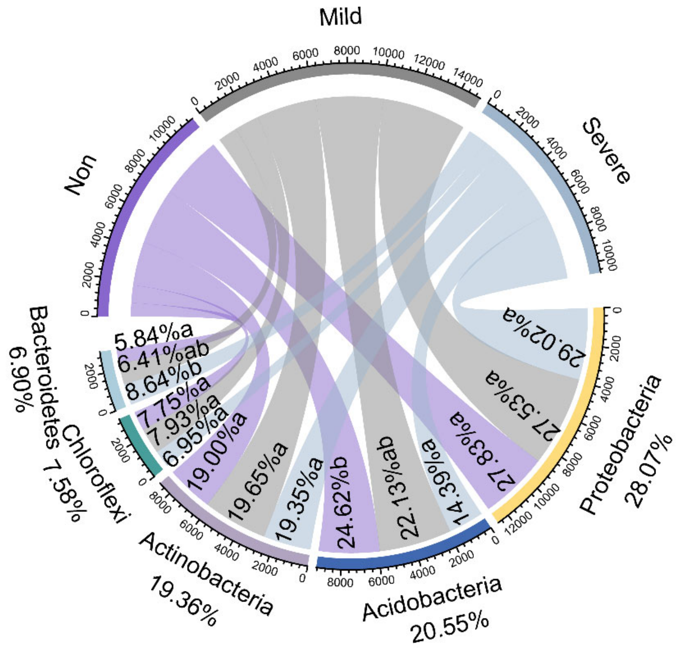

Figure S1. Phylogenetic distributions of dominant bacterial phyla in soils with three salinity levels, Non-salinity, Mild-salinity and Severe-salinity. Different letters denote significant phylogenetic differences ($p<0.05$) among salinity levels.

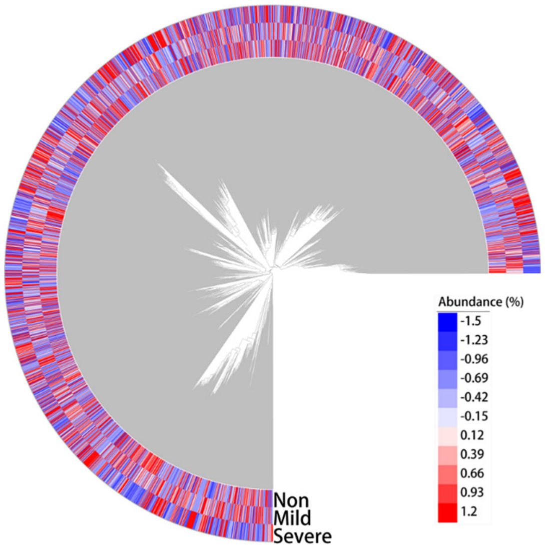

Figure S2. Phylogenetic distribution of the 3123 bacteria and their relative abundance among three salinity soils.
